# Supplementary material for: Atsttrin regulates osteoblastogenesis and osteoclastogenesis through the TNFR pathway
Source: Commun Biol. 2023 Dec 11;6:1251. doi: 10.1038/s42003-023-05635-y (PMC10713527; doi:10.1038/s42003-023-05635-y)
Supplement: Supplementary file 2 — Supplementary Material [file 42003_2023_5635_MOESM2_ESM.pdf]

# Atsttrin Regulates Osteoblastogenesis and Osteoclastogenesis through the TNFR Pathway

**Kaiwen Liu[1], Zihao Wang[1], Jinbo Liu[1], Wei Zhao[1], Fei Qiao[1,2], Qiting He[3], Jie Shi[1], Qunbo Meng[1], Jianlu Wei[1*] and Lei Cheng[1*]**

[1]Department of Orthopedic Surgery, Qilu Hospital, Cheeloo College of Medicine, Shandong University, Jinan, Shandong, 250012, China.

[2]Department of Pediatric Orthopedic, Dalian Women and Children′s Medical Center(group), Dalian, Liaoning, 116012, China.

[3]Department of Orthopedics, Honghui Hospital, Xian Jiaotong University, Xian, Shanxi, 710054, China

*** Correspondence:**

Corresponding Author：

Jianlu Wei, Email: 18560089157@163.com

Lei Cheng, Email: chenglei@email.sdu.edu.cn

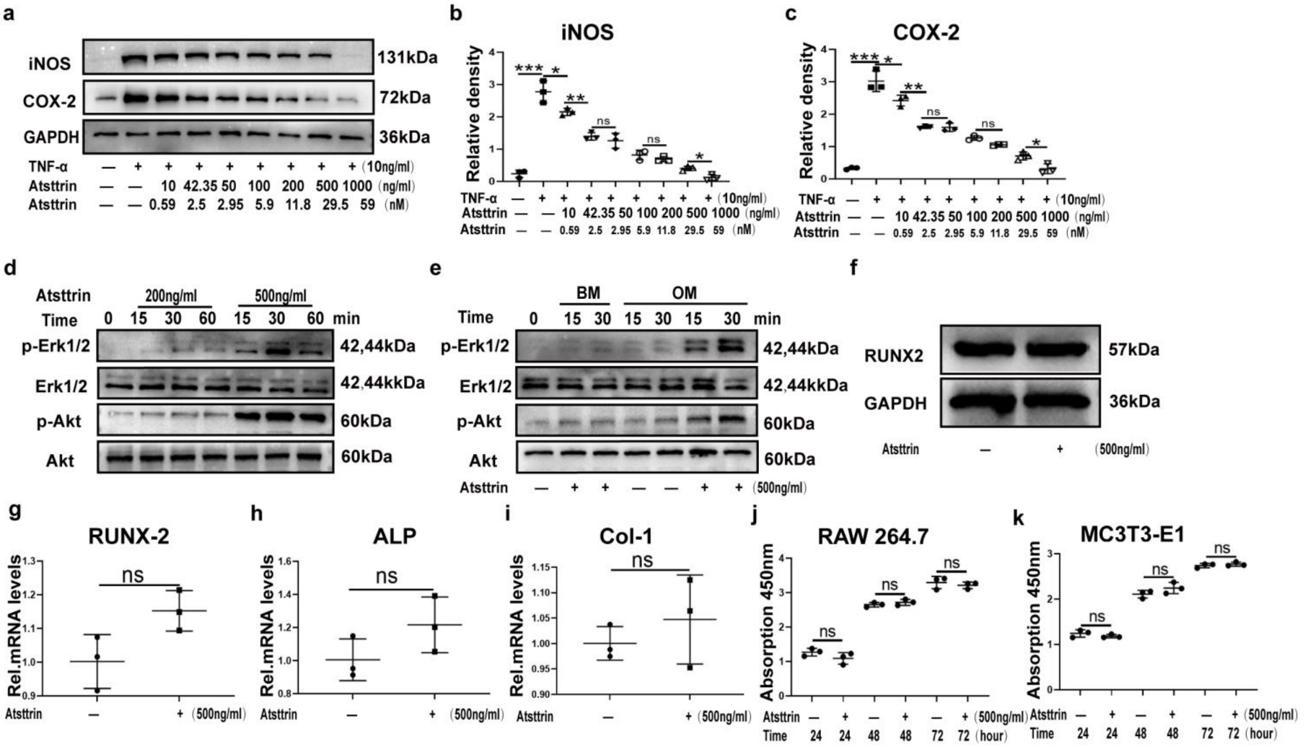


**Supplementary Figure 1. (a-c)** RAW264.7 cells were treated with TNF-α (Mass concentration: 10 ng/ml; Molar concentration: 0.588 nM) and various Atsttrin concentrations (Mass concentration：10 ng/ml, 42.35ng/ml, 50 ng/ml, 100 ng/ml, 200 ng/ml, 500 ng/ml and 1000 ng/ml; Molar concentration: 0.59 nM, 2.5nM, 2.95 nM, 5.9 nM, 11.8nM, 29.5 nM, 59 nM) for 48 hours. Inflammatory marker levels; iNOS and COX-2 were tested by Western blot. The expression of proteins mentioned above was quantitatively analyzed related to GAPDH (n=3). **(d)** MC3T3-E1 cells were treated with Atsttrin (200ng/ml, 500 ng/ml) for various time points, total protein was examined by Western blot. The bands in the figure are not all derived from the same membrane. **(e)** MC3T3-E1 cells were treated with Atsttrin (500 ng/ml) in absence or presence of osteogenesis-induced culture medium, and collected at various time points, followed by Western blot analysis (n=3). BM, basic medium. OM, osteoblastogenesis medium. The bands in the figure are not all derived from the same membrane. **(f)** MC3T3-E1 cells were treated with Atsttrin (500 ng/ml) in basic medium for 48 hours. Western blot to confirm RUNX2′s expression (n=3). **(g-i)** MC3T3-E1 cells were treated with Atsttrin (500 ng/ml) in basic medium for 8 hours. The mRNA expression levels of ALP, RUNX2 and Col-1 were tested by Real time PCR (n=3). **(j-k)** RAW264.7 cells and MC3T3-E1 cells were treated with or without Atsttrin (500 ng/ml) for 24, 48 and 72 hours, CCK-8 assay to test the effect on cell proliferation (n=3). Significant differences are indicated as follows: ∗P<0:05, ∗∗P<0:01 and ∗∗∗P<0:001.

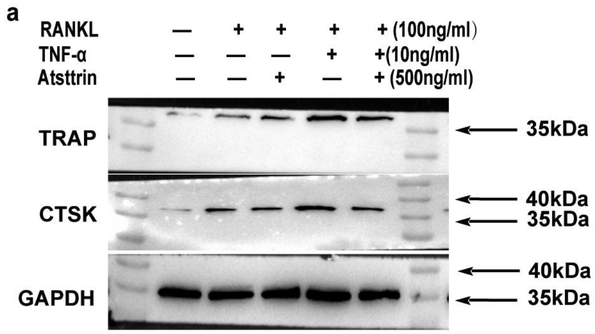


**Supplementary Fig 2. Related to Figure 1c. (a)** Western blotting analysis of osteoclastic marker (TRAP and CTSK) protein expression. Primary band images are indicated on top. GAPDH is used as a loading control. The bands in the figure are not all derived from the same membrane.

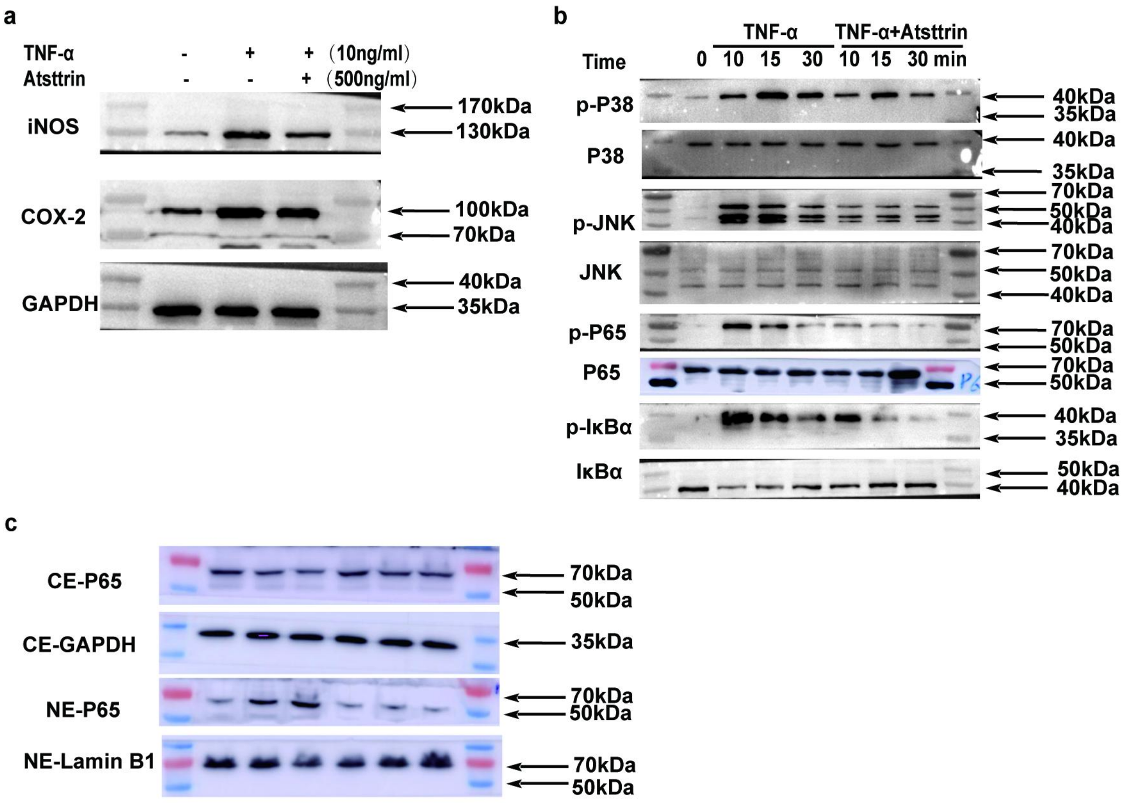


**Supplementary Fig 3. Related to Figure 2e, 2j, 2o. (a)** Representative images of Western blotting analysis of iNOS and COX-2 in RAW264.7 cells treated with TNF-α and Atsttrin. **(b)** Western blotting analysis of MAPK signaling pathway-related proteins (p-P38 and p-JNK) and NF-κB signaling pathway-related proteins (p-P65 and p-IκBα) expression. **(c)** Western blot analyses showed that p65 nuclear transfer levels. Primary band images are indicated on top. The Fig S 2b bands are not all derived from the same membrane.

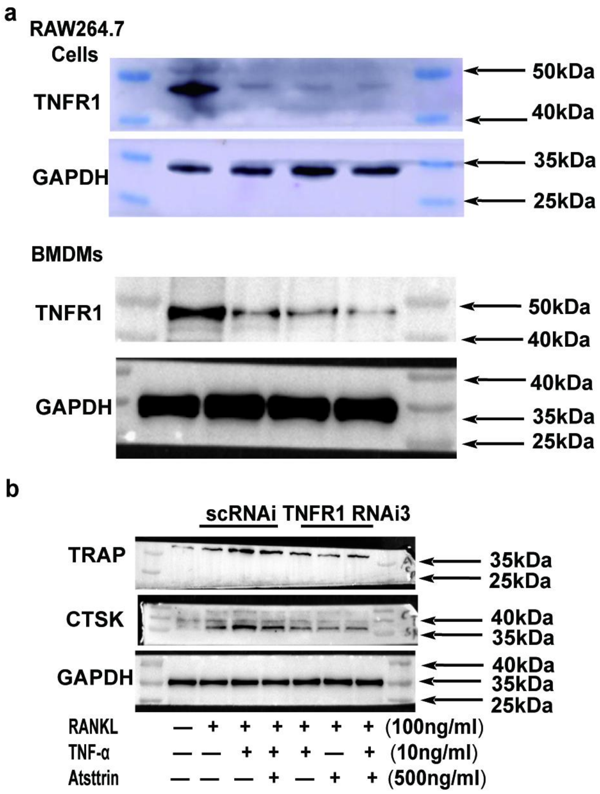


**Supplementary Fig 4. Related to Figure 3a, 3b. (a)** Representative images of Western blotting analysis of TNFR1 in RAW264.7 cells and BMDMs. **(b)** Western blotting analysis of the osteoclastic-related protein expression (TRAP and CTSK). Primary band images are indicated on top. GAPDH is used as loading control. The Fig S 4b bands are not derived from the same membrane.

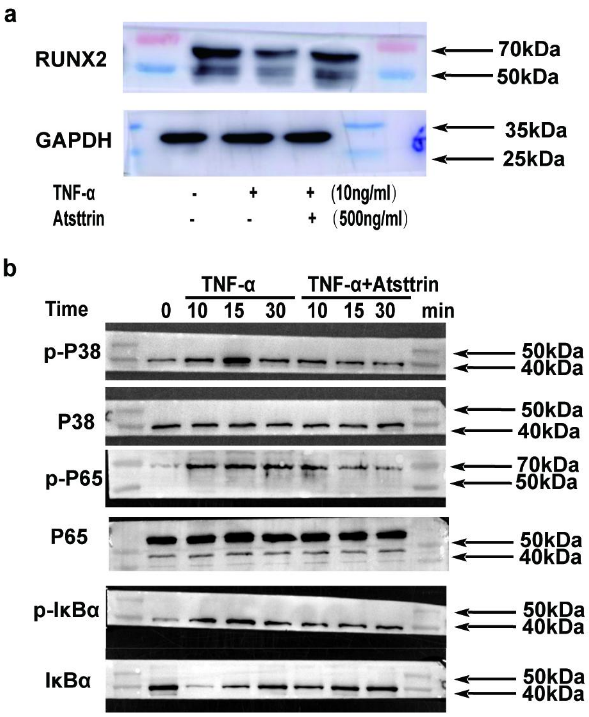


**Supplementary Fig 5. Related to Figure 4c, 4h. (a)** Western blotting analysis of the expression of the osteogenic markers RUNX-2. **(b)** As a result of Western blotting analysis, MAPK signaling pathway-related proteins (p-P38) and NFκB related proteins (p-P65 and p-IκBα) were tested. Primary band images are indicated on top. The Fig S 5b bands are not derived from the same membrane.

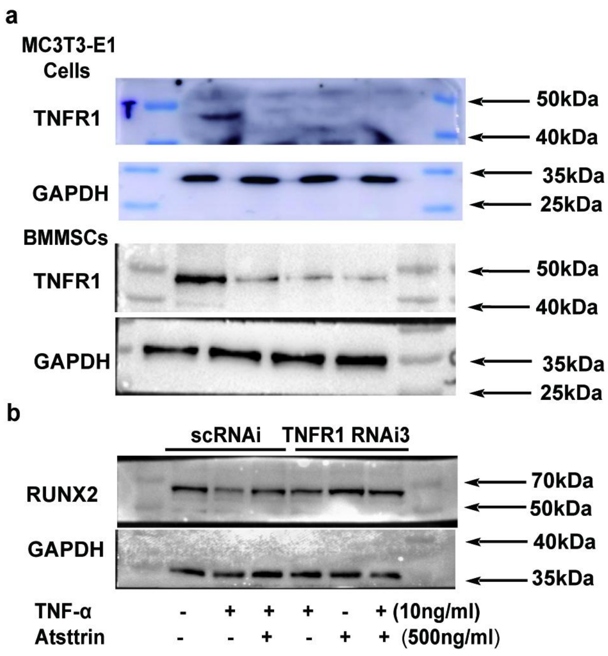

**Supplementary Fig 6. Related to Figure 5a, 5b. (a)** Western blot analyses showed TNFR1 levels in MC3T3-E1 cells and BMMSCs. **(b)** The WB showed that the protein expression of RUNX2. Primary band images are indicated on top. GAPDH is used as loading.

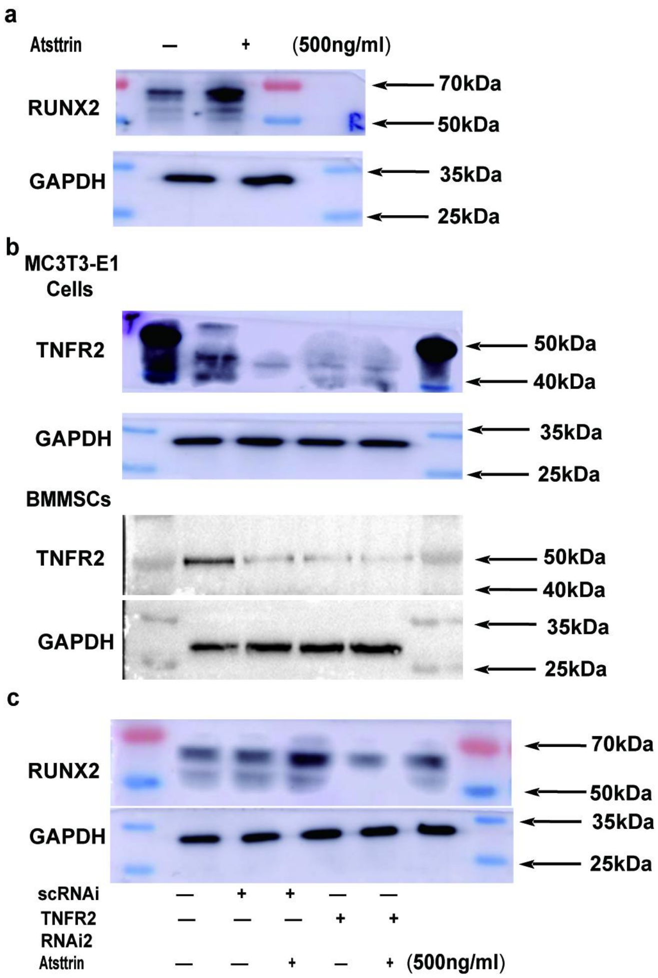


**Supplementary Fig 7. Related to Figure 6a, 6i, 6j. (a)** Western blotting analysis of RUNX2 protein expression. **(b)** Western blot analyses showed TNFR2 levels in MC3T3-E1 cells and BMMSCs. **(c)** The WB showed that the protein expression of RUNX2. Primary band images are indicated on top. GAPDH is used as loading.

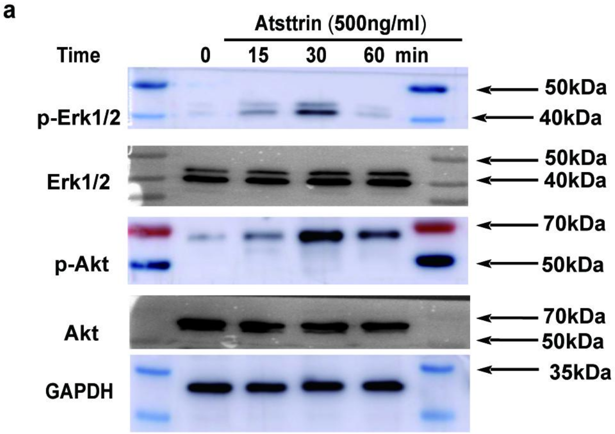


**Supplementary Fig 8. Related to Figure 7a. (a)** A western blot analysis was carried out on Atsttrin-induced MC3T3-E1 cells at different time points (15, 30 and 60h) with antibodies directed at p-AKT, AKT, p-ERK1/2, ERK1/2 and GAPDH. Primary band images are indicated on top. The bands in the figure are not all derived from the same membrane.

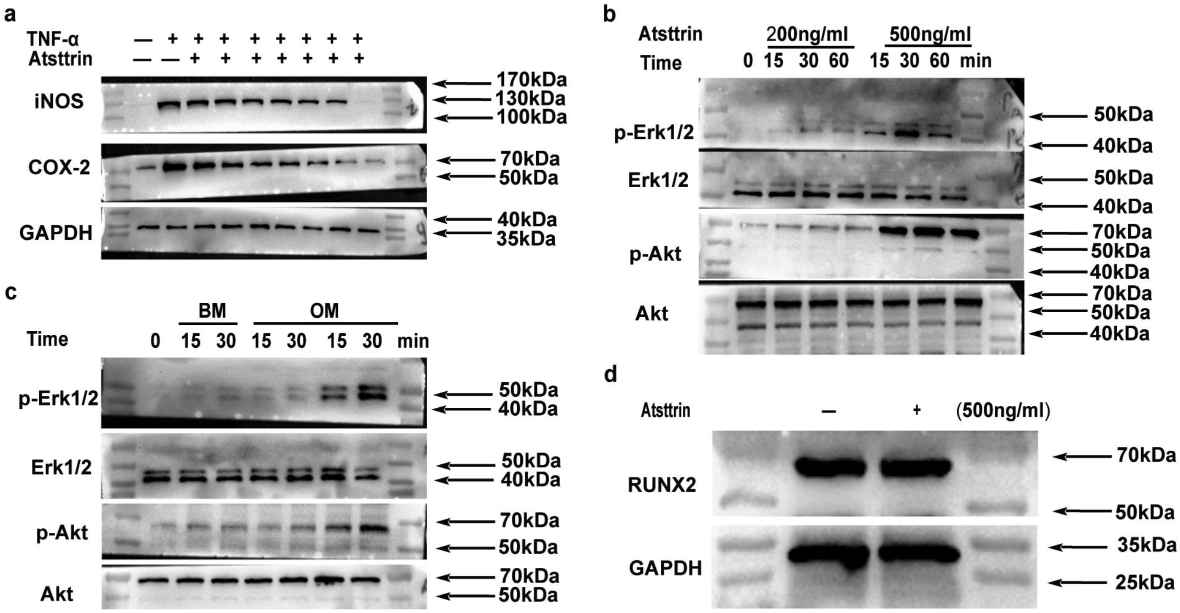

**Supplementary Fig 9. Related to Supplementary Figure 1a, 1d, 1e, and 1f. (a)** The WB showed the protein expression of iNOS and COX-2. **(b-c)** Western blotting analysis showed the protein expression of p-AKT, AKT, p-ERK1/2 and ERK1/2. **(d)** The WB showed that the protein expression of RUNX2. Primary band images are indicated on top. The Fig S 9b and 9c bands are not all derived from the same membrane.
