# Supplementary material for: Atsttrin regulates osteoblastogenesis and osteoclastogenesis through the TNFR pathway
Source: Commun Biol. 2023 Dec 11;6:1251. doi: 10.1038/s42003-023-05635-y (PMC10713527; doi:10.1038/s42003-023-05635-y)
Supplement: Supplementary file 3 — Description of Additional Supplementary Files [file 42003_2023_5635_MOESM3_ESM.pdf]

Description of Additional Supplementary Files

File name: Supplementary Data 1
Description: Source data behind the graphs in the figures.
